# Psychogenic or neurogenic origin of agrammatism and foreign accent syndrome in a bipolar patient: a case report

**DOI:** 10.1186/1744-859X-6-1

**Published:** 2007-01-04

**Authors:** Stéphane Poulin, Joël Macoir, Nancy Paquet, Marion Fossard, Louis Gagnon

**Affiliations:** 1 Centre de recherche Université Laval Robert-Giffard, 2601, rue de la Canardière Beauport (Qc), G1J 2G3, Canada; 2 Université Laval, Faculté de médecine, Pavillon Ferdinand-Vandry, Québec, (Qc) G1K 7P4, Canada; 3 Service de médecine nucléaire, Hôtel-Dieu de Lévis, 143, rue Wolfe, Lévis (Qc) G6V 3Z1, Canada

## Abstract

**Background:**

Foreign accent syndrome (FAS) is a rare speech disorder characterized by the appearance of a new accent, different from the speaker's native language and perceived as foreign by the speaker and the listener. In most of the reported cases, FAS follows stroke but has also been found following traumatic brain injury, cerebral haemorrhage and multiple sclerosis. In very few cases, FAS was reported in patients presenting with psychiatric disorders but the link between this condition and FAS was confirmed in only one case.

**Case presentation:**

In this report, we present the case of FG, a bipolar patient presenting with language disorders characterized by a foreign accent and agrammatism, initially categorized as being of psychogenic origin. The patient had an extensive neuropsychological and language evaluation as well as brain imaging exams. In addition to FAS and agrammatism, FG also showed a working memory deficit and executive dysfunction. Moreover, these clinical signs were related to altered cerebral activity on an FDG-PET scan that showed diffuse hypometabolism in the frontal, parietal and temporal lobes bilaterally as well as a focal deficit in the area of the anterior left temporal lobe. When compared to the MRI, these deficits were related to asymmetric atrophy, which was retrospectively seen in the left temporal and frontal opercular/insular region without a focal lesion.

**Discussion:**

To our knowledge, FG is the first case of FAS imaged with an ^18^F-FDG-PET scan. The nature and type of neuropsychological and linguistic deficits, supported by neuroimaging data, exclude a neurotoxic or neurodegenerative origin for this patient's clinical manifestations. For similar reasons, a psychogenic etiology is also highly improbable.

**Conclusion:**

To account for the FAS and agrammatism in FG, various explanations have been ruled out. Because of the focal deficit seen on the brain imaging, involving the left insular and anterior temporal cortex, two brain regions frequently involved in aphasic syndrome but also in FAS, a cerebrovascular origin must be considered the best explanation to account for FG's language deficits.

## Background

Foreign accent syndrome (FAS) is a rare speech disorder characterized by the appearance of a new accent, different from the speaker's native language and perceived as foreign by the listener and, in most cases, by the speaker also. Previous exposure to the new accent is not necessary for its emergence. Different explanations of the functional origin of FAS have been suggested, one of the more frequent being impaired access to verbal-motor patterns or a mild form of apraxia of speech. Clinical manifestations are heterogeneous among FAS patients but usually include segmental (e.g., changes in vowel length and tenseness) and prosodic (e.g., inappropriate word and sentence stress) deficits. Very few cases (n = 40) have been reported since the first descriptions of the syndrome by Pierre Marie in 1907 and Pick in 1919 [1]. It most often follows stroke and then overlays the recovery phase of non-fluent aphasia though it could persist beyond this phase. FAS has also been described following traumatic brain injury, cerebral haemorrhage and multiple sclerosis [2-7]. In a recent paper, Edwards, Patel and Pople [2] reviewed 35 case studies of FAS and showed that in 26 of them, the syndrome resulted from cerebral infarct, while 9 were due to head injury (6 cases), multiple sclerosis (2 cases) or episodes of psychosis (1 case). In 34% of these cases, FAS was also associated with agrammatism. Agrammatism is a frequent symptom of Broca's aphasia characterized by a deficit in sentence production. In spontaneous speech, agrammatic patients speak non-fluently and produce telegraphic speech. They mainly use content words (nouns, verbs, adjectives) and tend to omit or substitute function words (prepositions, articles and auxiliaries) as well as inflections or other grammatical morphemes. Among reported FAS cases, few brain imaging studies have been done and there is no consensus regarding the precise region responsible for its occurrence. Neuroanatomically, the vast majority of the lesions described were in the dominant hemisphere and in most cases involved regions typically associated with Broca's aphasia. Subcortical structures seem to be consistently affected [8].

Of all the reported FAS cases, very few (n = 3) cannot be clearly related to a neurological event, revealed by clinical exams and/or structural brain imaging studies [9-11]. For two of these cases, a psychological origin was never suggested although they were notable for psychiatric disorders [9,10]. In the third case, given the normal functional brain imaging results, conversion disorder was suggested as an explanatory mechanism [11].

This paper reports the case of FG, a bipolar patient presenting with language disorders characterized by a foreign accent and agrammatism initially categorized as being of psychogenic origin. Psychiatric patients do not commonly manifest speech or language disorders except when acutely psychotic. On formal language testing, schizophrenic and bipolar patients may present semantic verbal fluency and word finding difficulties when compared to controls [12]. To our knowledge, there are no instances of FAS and agrammatism previously reported in a bipolar patient.

## Case presentation

FG is a 74-year-old right-handed man. He has a grade eleven education and worked as an auxiliary nurse. He had suffered from a chronic bipolar disease since 1982, with multiple episodes requiring many hospitalizations. He came to our attention in July 2005 for acute exacerbation of a bipolar disorder with suspected psychotic features requiring inpatient treatment. At admission, symptoms were compatible with manic exacerbation. Psychotic features were not confirmed. Mental status examination revealed signs of his primary psychiatric disorder. Moreover, a foreign accent, English-sounding, was noted. FG had learned to deal with this long-lasting symptom so he did not report it spontaneously. However, on explicit questioning, he reported that this accent was socially invalidating and completely impossible to control or repress. FG is a native speaker of Quebec French but people who met him thought he came from somewhere else, most often Acadia (French-speaking areas of Eastern Canada (New Brunswick, Prince Edward Island and Nova Scotia) where the accent is markedly different from Quebec French), France or an English-speaking foreign country. Apart from this foreign accent, he also reported some German- or Spanish-sounding words occasionally and spontaneously coming to his mind. No meaning is associated with these words and the patient easily controls their occurrence with no anxiety. Neurological examination completed during the index hospitalization was unremarkable except for an observed inability to turn back on one foot (decomposition of the half-turn) when walking, slight incoordination of the left arm on the cerebellar testing, and slight micrographia. Snout and palmomental primitive reflexes were also noted.

FG's past medical records reported the presence of this foreign accent in January 2003. It was first noticed at the psychiatric outpatient clinic consultation, shortly after he was discharged from the inpatient service, which was required for manic exacerbation of his bipolar disorder in the fall of 2002. The presence of agrammatism was also recorded during the same period. Psychological factors were suspected because of the patient's psychosocial background (abuse by his father and emotional closeness to his mother, who was English-speaking). Even though he was exposed to English as a child, he never spoke or learned this language. Without any other neurological symptoms, his psychiatrist ascribed the foreign accent to a psychological phenomenon operating at an unconscious level.

His neurological history is noteworthy for epilepsy between the ages of 6 and 14 but without any other symptomatic seizures thereafter. He also suffered from delirium due to lithium intoxication 6 months before the onset of the foreign accent. Finally he has been treated for an essential tremor for many years and has neurosensory hypoacusia. Otherwise, there was no prior history of stroke, cranial trauma or encephalitis. When he developed the language disorder, he was on stable doses of lithium, valproate, quetiapine and perphenazine.

Although they appeared approximately 3 years earlier, the functional origin of the FAS and agrammatism was explored in FG through an extensive neuropsychological and brain imaging study.

### Neuropsychological evaluation

Neuropsychological testing showed no impairment in tasks exploring orientation to time and space. FG's performance was normal on the task exploring concentration and selective attention [13]. He showed good face recognition and presented no clinical signs of visual agnosia [14]. There were no signs of unilateral neglect. Praxis abilities were well preserved [15]. FG performed normally on tasks exploring episodic memory. His performance was within the normal range for the three recalls of the DMS-48, a visual forced-choice recognition test [16], as well as for the pictorial recognition memory test and the short recognition memory test for faces [17]. The patient's short term memory was normal in the visuospatial modality (forward span = 5; backward span = 4) [18]as well as in the verbal modality (forward digit span = 4; backward digit span = 3; forward word span = 4). FG presented with deficits on tests exploring working memory and executive functions. He presented with a severe impairment on the interference condition of the Brown-Peterson task [19], a test that taps the ability to encode, maintain, and manipulate information in working memory (see Table 1). His performance on the Stroop Test [20], an instrument designed to evaluate inhibition abilities (i.e. inhibition of a habitual or more automatic response in favour of an unusual one), was influenced by interference. He obtained normal scores in the word reading and colour naming but his performance was impaired in the colour-word conditions. FG also showed abnormal performance on the Trail Making [21], a test exploring mental flexibility (ability to manage more than one stimulus at a time and to shift the course of an ongoing activity). He made no mistakes but was slow on part A and his performance was poor on Part B (alternated switching between numbers and letters). Finally, FG's performance was impaired (2 SD below the normal range) on the D-Kefs Tower Test [22], a complex task that measures the executive functions of spatial planning, rule learning, and inhibition of impulsive responding.

**Table 1:** Performance of FG and norms (mean and standard deviation or range) on neuropsychological and language tests

**Test**	**FG's score**	**Norm**
Working memory and executive functions		
- Brown-Peterson test		
- no interference	100%	98.33% (4.47)
- mean of interference scores	42%*	97.22% (4.46)
- Stroop Test		
- Color name reading	74 sec.	48.5 sec (25–86)
- Color naming	105 sec.	69.4 sec (46–123)
- Interference	249 sec.*	142.4 sec. (88–204)
- Trail making test		
- Part A	61 sec.*	41.3 sec. (15)
- Part B	253 sec.*	111.4 sec. (72.2)
Language		
- Picture naming (DO80)	72/80	74.9 (2.94)
- Letter fluency (PENO)	5*	45.46 (16.4)
- Category fluency (PENO)	14*	47.85 (9.8)
- Pyramids and Palm Trees Test	47/52	49.4 (1.74)
- Token test	29/36	29–36
- Spoken word/sentence-to-picture matching (PENO)	44/47	44.6 (2.19)
- Written word/sentence-to-picture matching (PENO)	12/12	10.81 (.81)

* Indicates a score below the norm or out of the normal range

### Language evaluation

With regard to language, speech output was fluent and well articulated, with no signs of word-finding difficulties. The patient however presented with mild agrammatism. There were no phonemic or verbal paraphasias but speech was sometimes telegraphic with omissions of function words and grammatical bound morphemes as well as impoverished syntactic structure. Auditory and visuo-verbal input components were largely preserved. Comprehension abilities at the lexical-semantic level [23] as well as at the syntactic-semantic level [15,24] were normal (see Table 1). Reading and immediate and delayed repetition were flawless for both words and nonwords. Written spelling of nonwords was flawless but the patient's performance on word writing to dictation was canonical of surface agraphia with exclusive production of phonological plausible errors and performance affected by orthographic regularity and lexical frequency. However, the patient did not completely master the written language so that these results cannot be interpreted as actual deficits. FG's performance was normal in confrontation naming [25] but he showed difficulties in letter and semantic category fluency tasks [15] (see Table 1), a performance that could be attributed to the deficit in executive functioning. FG showed many characteristics usually reported for FAS. There were no signs of dysarthria (no slow, slurred, groping or laboured articulation) or apraxia of speech (no dysfluency and no problems with phoneme sequencing) but acoustic analysis performed on speech samples recorded in Digital Audio Tape showed the presence of abnormalities at the segmental and suprasegmental levels. Unfortunately, we had no premorbid recording of the patient's speech. However, FG himself as well as one of his close friends, who has known him for over 30 years, confirmed that he never had this particular strange accent before its sudden appearance in January 2003.

### Neuroimaging

**Figure 1:**
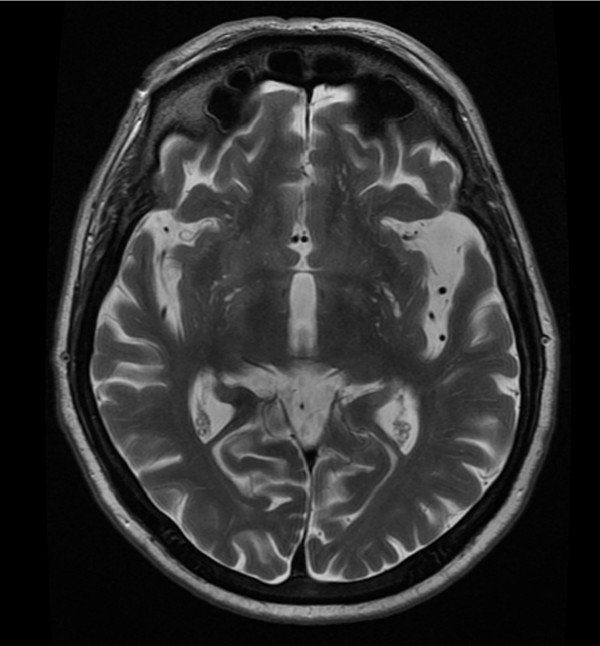
**Magnetic resonance imaging**. Axial T2-weighted sequence showing diffuse cortical atrophy predominating at the left sylvian fissure.

Neuroimaging studies were performed while the patient was in euthymic condition. A magnetic resonance imaging (MRI) study including sagittal FLAIR and T2-weighted sequences and axial FLAIR, proton density, T1 and T2-weighted sequences was performed on December 8, 2005 using the standard protocol. The first interpretation was normal except for slight diffuse cerebral atrophy considered normal for his age (see Figure 1: serie 3 31/10 = axial T2-weighted sequence showing diffuse cortical atrophy predominating at the left sylvian fissure).

An ^18^F-fluorodeoxyglucose brain positron emission tomography was obtained with a dual-head coincidence camera (Vertex MCD-AC, Phillips). After a 30-minute rest, 111 MBq ^18^F-FDG were injected in a veinous catheter. There was another 30-minute rest before starting the acquisition (64 × 64 × 16 matrix, 64 steps, mean of 25 seconds/step with decay correction). Measured attenuation and scatter correction were applied to the iterative reconstruction method.

**Figure 2:**
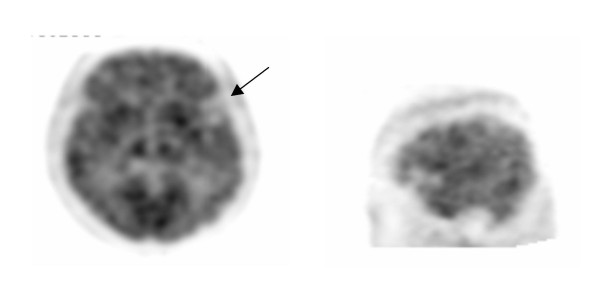
**Brain positron emission tomography**. ^18^F-FDG PET showing focal deficit in the area of the anterior left temporal lobe with proheminence of the sylvian sulcus.

The reconstructed images showed diffuse hypometabolism in the frontal, parietal and temporal lobes bilaterally whereas the cerebellum, occipital lobe and subcortical structures were spared. There was also a focal deficit in the area of the anterior left temporal lobe with prominence of the sylvian sulcus (see Figure 2). When compared to the MRI, these deficits were related to asymmetric atrophy, which was retrospectively seen in the left temporal and frontal opercular/insular region without a focal lesion.

## Discussion

We have reported the case of FG, a bipolar patient who presented with a sudden onset of FAS and agrammatism. He also showed a working memory deficit and executive dysfunction. These clinical signs were related to altered cerebral activity on the FDG-PET scan.

In FG, the FDG-PET scan is characterized by more diffuse hypometabolism and by marked hypometabolism in the area of the left insular and anterior temporal cortex. Functional neuroimaging revealed focal deficit signs while the MRI structural neuroimaging was initially considered a normal variant for FG's age. The MRI scan showed slight diffuse cerebral atrophy and an absence of indirect signs of vascular pathology such as hyper intense signals on T2-weighted images elsewhere in the brain. Retrospective analysis of the MRI scan showed the same asymmetry as noted on the PET scan, albeit less obviously. To our knowledge, FG is the first case of FAS imaged with an ^18^F-FDG-PET scan.

These structural and functional neuroimaging characteristics differ substantially from what was previously reported for bipolar disorder patients as a group. In fact, structural neuroimaging studies do not typically show overall brain volume loss but specific regional cerebral volume variations. Compared to controls, volume reductions in the subgenual cortex and cerebellar vermis, associated with enlargement in the striatum and amygdala, are usually noted in bipolar patients [26]. Unlike FG, no previous study showed insular cortex or anterior temporal cortex reduction; on the contrary, one showed an increase in the left insular/frontoparietal operculum cortex [27].

Despite variations in approaches (PET, SPECT, fMRI), paradigms used (at rest vs. while completing cognitive tasks), mood states studied (depressive, manic, euthymic) and treatment status (on mood stabilizers or not), converging results have been reported on functional neuroimaging of bipolar disorders [26]. Decreased metabolism and perfusion in the prefrontal cortex and particularly in the subgenual portion of the cingulated gyrus and striatum are observed during depressive phases in bipolar patients. Conversely, increased orbitofrontal cortex and cingulate gyrus activity along with their related subcortical structures including the striatum and thalamus is reported in manic states. Only one functional imaging study of euthymic bipolar patients at rest is reported in the literature. In that study, a state-dependent activation of the anterior part of the temporal lobe was observed for depressive/dysphoric and manic states. While euthymic, no altered temporal lobe activity was seen [28]. As a whole, these results do not indicate that FG's bipolar status may explain the altered functional imaging results. In this patient, it is more likely that the language disorders (FAS and agrammatism) are the external manifestation of the marked hypometabolism of the left insular and anterior temporal cortices.

The insula is frequently involved in major aphasic syndromes and especially in Broca's aphasia. This type of aphasia is caused by large lesions that damage the posterior lateral frontal lobe, including the operculum, anterior superior insula, anterior parietal lobe, and the white matter deep inside these structures. Lesions of the insula are associated with impairments in speech production and more specifically with articulatory planning deficits (i.e., apraxia of speech) [29]. This finding has received further support from neuropsychological [29] and neuroimaging studies [30]. The insula is also involved in sentence processing (comprehension and production). Moreover, patients with a lesion restricted to Broca's area usually did not produce agrammatic speech [31]. Larger lesions of the frontal and parietal opercula and the insula were required. A previous case of FAS showed perfusion deficits on a SPECT study in the regions of the left frontal motor cortex extending to the insula and subcortical structures in addition to the left anterior temporal lobe [4].

Executive dysfunction could also represent a clinical manifestation of the altered metabolism of the left insular cortex. Executive functions represent several higher level cognitive processes enabling adaptation to new or complex situations. Traditionally considered abilities relying on frontal lobes, the neural networks that underlie executive functions are now largely identified though not completely elucidated. They are probably specific, with each recruiting various cortical areas of the brain, not only in the frontal lobes but also in the parietal and temporal lobes as well as the cerebellum [32,33]. Subcortical structures play a critical role in executive functions. Insular cortex neural activity assessed by functional imaging was correlated with deficits in executive functions in several studies including normal [34-36] and clinical samples [37,38]. FG is treated with a typical antipsychotic (perphenazine) and shows clinical signs of parkinsonism (decomposition of the half-turn, micrographia) so that executive dysfunction could be related to basal ganglia impairment.

To account for FG's deficit, a possible neurotoxic origin must be considered. With respect to the previous lithium intoxication, delayed onset manifestation has never been reported. Except for dysarthria, speech disorders are rare in lithium neurotoxicity. Antipsychotics have never shown consistent alteration of language and cognitive functioning in clinical populations [39].

Because of the acute onset and stability of the symptoms in FG, the presence of a neurodegenerative process is highly improbable but should also be examined. Except for cognitive function deficits, none of the DSM-IV-TR [40] criteria for the diagnosis of dementia was met in FG. He showed no episodic memory problems, no agnosia, no apraxia, and his language difficulties did not correspond to what is usually encountered (i.e., word-finding and comprehension problems) in the early phase of major forms of dementia. Moreover, the patient's cognitive impairment had no impact on his social participation and activities of daily living. FG presented with abnormalities in the left anterior temporal lobe, a cortical localization compatible with frontotemporal dementia (FTD). However, except for executive function deficits, the patient's clinical profile did not meet the clinical diagnosis features of FTD [41]. Apart from episodes of decompensation, he presented neither character change nor disordered social conduct, the dominant features at the onset of and throughout the course of FTD. With respect to language, he did not show any of the supportive diagnosis features of FTD (aspontaneity, echolalia, perseveration, etc). Finally, progressive nonfluent aphasia (PNFA) is a clinical syndrome associated with FTD [41] in which agrammatism is sometimes observed [42]. However, FG did not present any of the PNFA core diagnostic features (nonfluent spontaneous speech, phonemic paraphasias, anomia). Moreover, FAS has never been reported in PNFA, as in any other forms of dementia.

In FG's case, conversion disorder must be excluded as the primary mechanism responsible for the foreign accent and agrammatism. Speech disorders of conversion origin typically present as dysarthria, mutism, aphonia or stuttering [43]. Foreign accent and agrammatism would be a rather unusual presentation of conversion disorder. Furthermore, FG had never heard of or known anyone suffering from this disorder before it appeared in 2003, making unconscious mimicry almost impossible. Nevertheless, conversion disorder may superimpose on complex neurological symptoms, giving them chronic course [44]. Therefore, it cannot be totally ruled out that conversion mechanisms contribute to the foreign accent and agrammatism in FG. A typical chronic FAS has recently been ascribed to conversion disorder [11]. For now, because of the way the DSM-IV-TR criteria are formulated, there is no way to convincingly exclude a conversion disorder contribution to a neurological symptom of unknown origin [40]. In fact, conversion disorder is the only DSM-IV-TR diagnosis that includes in its definition criteria a putative causative mechanism which, in any event, can never be ruled out. Therefore, because of the absence of clear and specific criteria, a diagnosis of conversion disorder is automatically considered when there is no alternative hypothesis. For the moment, functional brain imaging as well as electrophysiological studies cannot help either. These types of studies have shown alterations of specific brain regions in neurological dysfunction of conversion origin [45,46].

## Conclusion

Initially attributed to a psychogenic phenomenon, the origin of FAS and agrammatism in FG is now clearer. Different functional origins were considered and have been largely ruled out. Neither FAS nor agrammatism have been reported in bipolar disorder patients. Moreover, neuroradiological correlates in these patients usually differ from what was observed in FG. The nature and type of clinical manifestations also exclude a neurotoxic or neurodegenerative origin for FG's cognitive symptoms. For similar reasons, a conversion disorder also appears to be a highly improbable etiology even though a possible contribution cannot be totally excluded. Because of the focal deficit seen on the brain imaging, involving the left insular and anterior temporal cortex, two brain regions frequently involved in aphasic syndrome but also in FAS, a cerebrovascular origin must be considered the best explanation to account for FG's language deficits. We therefore conclude that in this patient, as in few other reported cases, the FAS is associated with agrammatism as a direct consequence of a cerebral infarct.

## Competing interests

The author(s) declare that they have no competing interests.

## Authors' contributions

SP contributed to the patient's care and referred him to JM for the clinical study. JM was the study coordinator. SP and JM reviewed the existing literature and drafted the manuscript. MF and NP reviewed the manuscript and contributed to the writing. NP and LG conducted the brain imaging exams and interpreted the data. All the authors approved the final manuscript.
